# Protective Effect of *Scutellaria litwinowii* Extract on Serum/Glucose-Deprived Cultured PC12 Cells and Determining the Role of Reactive Oxygen Species 

**DOI:** 10.1155/2012/413279

**Published:** 2012-07-19

**Authors:** Maryam Afsharzadeh, Zahra Tayarani-Najaran, Aryo Zare, Seyed Hadi Mousavi

**Affiliations:** ^1^Pharmacological Research Centre of Medicinal Plants, School of Medicine, Mashhad University of Medical Sciences, Mashhad, Iran; ^2^Department of Pharmacodynamics and Toxicology, School of Pharmacy, Mashhad University of Medical Sciences, Mashhad, Iran; ^3^Research Center of Natural Products Safety and Medicinal Plants, North Khorasan University of Medical Sciences, Bojnurd, 01830-49504, Iran; ^4^Medical Toxicology Research Center, School of Medicine, Mashhad University of Medical Sciences (MUMS), Mashhad, Iran

## Abstract

Considering the wide, positive reporting of the role of reactive oxygen species in ischemic brain injury, searching for antioxidant drugs within herbal remedies is logical. In this study, the protective effects of *Scutellaria litwinowii* Bornm. & Sint. on cell viability and reactive oxygen species production in cultured PC12 cells were investigated under serum/glucose-deprivation-induced cell death. After cells were seeded overnight, they were then deprived of serum/glucose for 24 h. Cells were treated with different concentrations of *S. litwinowii* extract (7.75–250 **μ**g/mL). Cell viability was quantitated by MTT assay, and intracellular reactive oxygen species production was measured by flow cytometry. Serum/glucose-deprivation induced significant cell death after 24 h (*P* < 0.001). Treatment with *S. litwinowii* (7.75–250 **μ**g/mL) reduced serum/glucose deprivation-induced cytotoxicity in PC12 cells after 24 h. A significant increase in intracellular reactive oxygen species production was seen following serum/glucose deprivation (*P* < 0.001). *S. litwinowii* (62 and 125 **μ**g/mL, *P* < 0.01) treatment reversed the increased reactive oxygen species production following ischemic insult. This demonstrates that *S. litwinowii* extract protects PC12 cells against serum/glucose-deprivation-induced cell death by antioxidant mechanisms, which indicates the potential therapeutic application of *S. litwinowii* in managing cerebral ischemic and neurodegenerative disorders.

## 1. Introduction

Despite considerable progress in stroke pharmacotherapy, ischemic and neurodegenerative cell death is still a major concern. As the underlying patho-physiology is decrementation of glucose, O2 and other micronutrients toward neurons, serum/glucose deprivation (SGD) can be used as a suitable model to evaluate stroke process and the effects of pharmacological remedies as discussed by Moley and Mueckler [1]. The PC12 rat pheochromocytoma cell line has been widely used as a representative in vitro model to analyze the different biochemical alterations in neural tissue as discussed elsewhere [2], thus making it a good model to analyze SGD conditions according to Woronowicz et al. [3].

*Scutellaria* is a subset of Lamiaceae, which has antioxidant effects as described previously by Martin and Dušek [4].

Millions of patients suffer yearly from ischemic insults to different organs, and it has been discovered that nearly all of them suffer from the detrimental effects of reactive oxygen species (ROS). In contrast, there are specific substances called antioxidants capable of scavenging and neutralizing these ROS; the most important of which are enzymes (e.g., superoxide dismutase, catalase, and glutathion peroxidase). There are also other nonfree radical substances, which possess plenty of reactivitymainly due to high-energy oxygen bonds (e.g., hydrogen peroxide, lipoperoxides and hydroperoxides and epoxides of endogen lipids, and hypochloric acid) so that the term ROS embraces several categories of substances other than free radicals. ROS production is particularly massive after reperfusion due to uncontrolled internal mitochondrial metabolism, arachidonic acid metabolism, and the resulting eicosanoid production by the lipoxygenase pathway. Cyclooxygenase is a major player of these detrimental cascades as well. There are also other peroxide compounds released by inflammatory leukocytes that add to the chaos after reperfusion. Large amounts of oxygen and the resulting increase of ATP production is followed by its rapid degradation and production of reactive oxygen metabolites by the xanthine oxidase pathway. This pathway gains much more importance during ischemia due to the conversion of xanthine dehydrogenase to xanthine oxidase. There is also activation of nitric oxide synthase (NOS) after increased intracellular Ca^2+^, which produces NO and is responsible for the subsequent conversion of it into a highly reactive metabolite ONOO^−^ that adds to its complexity during reperfusion injury [5, 6]. ONOO^−^ damages proteins, nucleic acids, and membrane phospholipids. Polyunsaturated fatty acids (PUFAs) within cell membranes are particularly sensitive to free radical oxidation mainly because of their highly reactive covalent bonds.

The lipid peroxidation cascade results in the production of alcoholic peroxide and hydroperoxide analogues of such fatty acids after reaction with Fe^2+^, which then produces toxic aldehye metabolites such as a crolein. These reactions ultimately result in decreased membrane liquidity, altered transmembrane enzyme and receptor systems, and finally the rupture of membranes. Effects upon proteins are even more important and include increased carbonyl groups, decreased sulfhydryl groups, increased disulfide bonds, reduced covalent amine bonds nitrolation of tyrosines, tryptophan breakdown, and the ultimately destruction of the 4th and 3rd configurations of proteins. Gene expression is altered after oxidative stress as large quantities of mRNAs are transcribed and subsequently translated to make nuclear factor kappa B (NF kappa B), which itself will alter numerous other genes (e.g., cyclooxygenase, inducible nitric oxide and metalloproteinase, intercellular connection molecules and cytokines). Overall there is a proportional increase of neuronal death either by necrosis or apoptosis after the generation of ROS. 

Neurons are much more sensitive to glucose deprivation than other tissues because they lack glycogen stores. The type of sensor motor deficit depends on the anatomical location of the insult and so displays vast diversity due to the complexity of the neuroanatomy of the region [7]. Today there is an increasing interest toward investigating the possibilities of herbal remedies in the prevention and treatment of ischemic and neurodegenerative cell death. *S. litwinowii *root is a traditional herb, reported to have antioxidant effects [4]. This study has attempted to elucidate the protective effects of *S. litwinowii *root extract and the antioxidant benefits of this root in reversing ROS increment and cell toxicity and to study the role of ROS production in GSD-induced PC12 cell toxicity. 

## 2. Materials and Method

### 2.1. Reagents

4, 5-Dimethylthiazol-2-yl, 2, 5-diphenyl tetrazolium (MTT), 2, 7-dichlorofluorescin diacetate (DCFH-DA), and Dulbecco's Phosphate-buffered saline (PBS) were purchased from Sigma (St Louis, MO, USA). Glucose-high Dulbecco's modified Eagle's medium (DMEM), Glucose-free DMEM, fetal bovine serum (FBS), and penicillin streptomycin were purchased from Gibco (Grand Island, NY). Dimethyl sulfooxide (DMSO) was purchased from Merck. The root of *S. litwinowii *was collected in its natural habitat in Hosseinabad valley (2100 m height) in Pivejan, a village 65 km southwest of Mashhad, Khorasan Razavi province, Iran, and was authenticated by the Ferdowsi University of Mashhad Herbarium. Voucher specimen was deposited in the Herbarium of the Faculty of Pharmacy, University of Mashhad Medical Sciences.

### 2.2. Preparation of *S. litwinowii* Methanolic Root Extract

*S. litwinowii *methanolic root extract was prepared as described previously by Tayarani-Najaran et al. [8]. Dry powdered roots (100 g) of *S. litwinowii *were extracted using methanol (4  ×  0.5 L) and were then concentrated at 50°C under reduced pressure to dryness. 

### 2.3. Cell Culture

PC12 cells were obtained from Pasteur Institute (Tehran, Iran). Cells were maintained at 37°C in a humidified atmosphere (90%) containing 5% CO_2_. Cells were cultured in high glucose Dulbecco's modified Eagle's medium (DMEM) (4.5 g/L Glucose) with 10% (v/v) fetal bovine serum, 100 units/mL penicillin, and 100 *μ*g/mL streptomycin.

For the experiments, PC12 cells were seeded overnight and then were subjected to serum/glucose deprivation for 24 h by replacing the culture medium with the glucose-free DMEM supplemented with 100 U/mL penicillin, and 100 U/mL streptomycin. PC-12 cells were then pretreated (2 h) with *S. litwinowii *extract (7.75–250 *μ*g/mL) and subjected to serum/glucose deprivation (SDG) for 24 h. For MTT assay, cells were seeded at 5000/well onto 96-well culture plates. For assay of ROS production, cells were seeded at 100'000/well onto a 24-well plate. For each concentration and time course study, there was a control sample remained untreated and received the equal volume of medium. 

### 2.4. Cell Viability

The cell viability was determined using a modified 3-(4,5-dimethylthiazol-2-yl)-2,5-diphenyl tetrazolium (MTT) assay [9]. Briefly, they were seeded (10^4^ cell/well) onto flat-bottomed 96-well culture plates. After removing the medium, MTT solution (5 mg/mL in PBS) was added for 1.5 h resulting formazan, which was solubilized with DMSO (100 *μ*L). The absorption was measured at 570 nm (620 nm as a reference).

### 2.5. Measurement of Intracellular Radical Oxygen Species

The determination of intracellular radical oxygen species (ROS) levels was accomplished as described previously with minor modifications [10, 11]. In brief, at 18 h after ischemic insult, the PC12 cells were incubated with 10 *μ*M DCFH-DA at 37°C for 30 min in the dark. The fluorescence of 2′,7′-dichlorofluorescein (DCF), the oxidation product of DCFH-DA, was excited at 480 nm and detected at 530 nm by flow cytometry. Temperature was maintained at 37°C throughout the experiment. 

### 2.6. Statistics

One-way analysis of variance (ANOVA) was used for data analysis. All results were expressed as mean ± SEM. *P* < 0.05 was considered statistically significant. 

## 3. Results

### 3.1. *S. litwinowii* Extract Protected PC12 Cells against Serum/Glucose Deprivation-Induced Cytotoxicity

**Figure 1:**
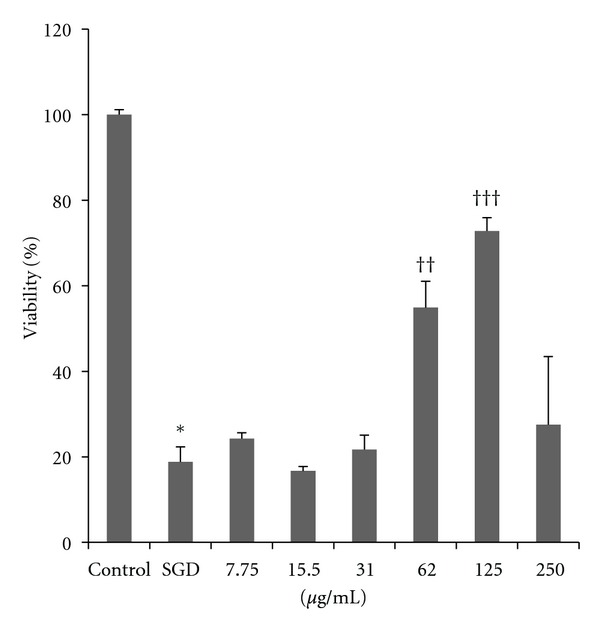
Protective effects of *S. litwinowii *on serum/glucose deprivation-induced cytotoxicity in PC12 cells. PC12 cells were pretreated with *S. litwinowii *(7.75–250 *μ*g/mL) for 2 h and then were exposed to serum/glucose deprivation for an additional 24 h with respective original concentrations of *S. litwinowii*. The incubation in the high-glucose medium during the whole treatment period served as control group, and the treatment only with serum/glucose-free medium for 24 h served as serum/glucose deprivation alone group. The cell viability was expressed as the percent (%) of the control value by using MTT assay. The data presented are means ± SEM of three independent experiments (*n* = 3). *: *P* value <0.001 relative to control group; ^††^: *P* value <0.01 relative to SGD; ^†††^: *P* value <0.001 relative to SGD.

The result showed that serum/glucose deprivation could decrease cell viability of cultured PC12 cells in a time-dependent manner compared to control (Figure 1). Exposure to serum/glucose deprivation for 6, 18, and 24 h decreased cell viability to 41.7 ± 2.37%, 33.5 ± 1.53%, and 18.8 ± 3.52%, respectively (*P* < 0.001). 24 h serum/glucose deprivation was then selected to induce PC12 cell injury in all subsequent experiments. The results of this study show that SGD had a destructive effect upon cells, which was shown both morphologically and by MTT assay. This resulted in a cell viability of less than 20%; however, by adding *S. liwinowii* root extract, this destructive process was significantly reversed at 62.5 and 125 *μ*g/mL (Figure 1).

**Figure 2:**
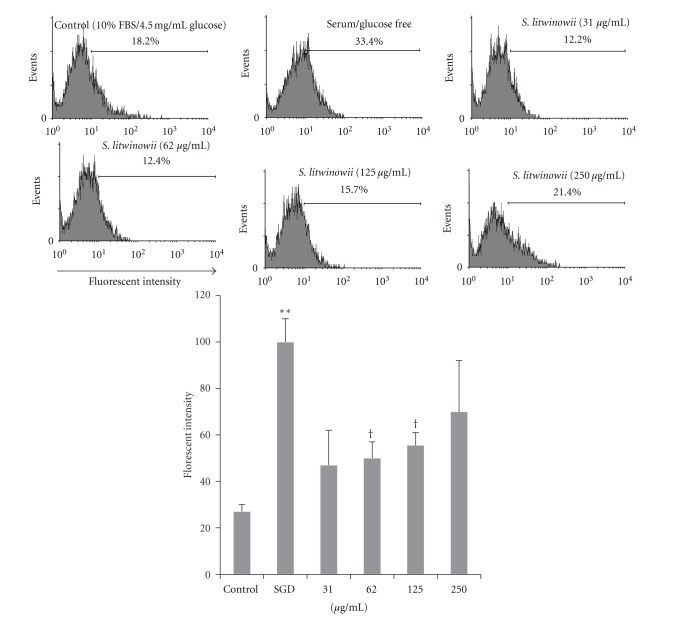
Flow cytometry with DCFH-DA staining for measuring ROS production for cultured PC12 cells in control group, serum/glucose deprivation alone group, and pretreatment with *S. litwinowii *(15–250 *μ*g/mL) plus exposure to serum/glucose deprivation. In the pretreatment groups, the PC12 cells were exposed to serum/glucose deprivation in the presence of *S. litwinowii* (15–250 *μ*g/mL) for only 24 h. The ROS production was assessed according to changes in the fluorescence intensity of DCF, the oxidation product of DCFH-DA. The data presented are means ± SEM of three independent experiments (*n* = 3). *: *P* value <0.001 relative to control group; ^†^: *P* value <0.05 relative to SGD.

Pretreatment with *S. litwinowii *extract (7.75–250 *μ*g/mL) at different concentrations significantly attenuated SGD-induced toxicity in PC12 cells. Consequently, concentrations at around 62.5 and 125 *μ*g/mL can be considered as protective (Figure 2). There were no significant toxic effects when PC12 cells were incubated with *S. litwinowii *extract (7.75–250 *μ*g/mL) (Data were not shown).

As shown in Figure 2, the less toxicity was seen following pretreatment of PC12 cells with 125 *μ*g/mL *S. litwinowii *extract. There was also a detrimental effect at 250 *μ*g/mL, which reveals initiation of cell toxicity at such a concentration.

### 3.2. Effects of *S. litwinowii* Extract on Serum/Glucose Deprivation-Induced Elevation of ROS Production in PC12 Cells

Molecular probe DCFH-DA was used to monitor alterations in intracellular ROS levels with flow cytometry. To elucidate the antioxidant effects of the extract, cells were exposed to stressful conditions after being treated with *S. litwinowii *extract (31–250 *μ*g/mL) and fluorescence intensity was measured.

ROS production was measured following the exposure of PC12 cells to serum/glucose deprivation for 24 h with or without the pretreatment with *S. litwinowii *extract (31–250 *μ*g/mL). As shown in Figure 2, serum/glucose deprivation for 24 h could significantly increase the number of DCF-positive cells illustrating an elevation of ROS production compared to control. Flow cytometry Analysis was performed by WinMDI software. The vertical axis is the fluorescence intensity, which is proportional to the cells ROS content, while the horizontal axis distinguishes the cell count. Figure 2 shows that pretreatment with same concentrations, which increased cell viability of* S. litwinowii *extract (62.5 and 125 **μ**g/mL), resulted in a significant attenuation of ROS production following SGD (Figure 2).

## 4. Discussion

Considering the high economic and social burden of ischemic brain injury and neurodegenerative diseases, research into their causes and prevention is vital. In modern medicine, the most important means of halting ischemic injury is through pharmacotherapy, either in synthetic or herbal forms. Clearly, studies into the means of herbal remedies in treating and preventing diseases and of their behavior can lead to positive outcomes. *S. litwinowii *is an endemic herb of Iran. In a study by Tayarani-Najaran et al., its apoptogenic properties on cancer cell lines have been recently demonstrated [8]; however, studies to examine its antioxidant effects and its effect upon a neural cell line have yet to be undertaken. PC12 cell lines have been studied previously as a neural ischemic model, but those studies were conducted on synthetic drugs or other herbal remedies. There was a study on ROS production after hyperglycemic stress upon PC12 cell lines, but the study used saffron extract as a potential beneficial agent [11, 12]. The effect of ROS production in oxidative damage and the role of antioxidant therapy were assessed by several studies [13, 14]. Antioxidant pharmacotherapy has been vastly utilized in patients with ischemic brain injury and has been successful to a large extent as discussed by Gilgun-Sherki et al. [15]. This is the first study on the protective effects of *S. litwinowii *on PC12 cell lines.

In this study, initially the positive and negative effects of total *S. litwinowii *root extract by MTT assay were shown. There was a reduction of cell death after treatment with specific amounts of concentrations of the extract in cultured and conditioned PC12 cells. This indicates that there is an active substance with protective potential present in *S. litwinowii *root extract.

Acute occlusion of an intracranial vessel restricts blood flow to the brain region it supplies. The magnitude of flow reduction is a function of collateral blood flow, and this, depends on individual vascular anatomy and the site of occlusion. A fall in cerebral blood flow to zero causes death of brain tissue within 4–10 min; values <16–18 mL/100 g tissue per min cause infarction within an hour; and values <20 mL/100 g tissue per min cause ischemia without infarction unless prolonged for several hours or days. If blood flow is restored prior to a significant amount of cell death, the patient may experience only transient symptoms, that is, a transient ischemic attack. Tissue surrounding the core region of infarction is ischemic but reversibly dysfunctional and is referred to as ischemic penumbra. The penumbra may be imaged by using perfusion-diffusion imaging with MRI. The ischemic penumbra wills eventually infarct if there is no change in flow, and hence saving the ischemic penumbra is the goal of revascularization therapies. This penumbra is optimistically responsive to pharmacotherapy, including antioxidants treatment. Pharmacotherapy also provides opportunity for the patient to be prepared for revascularization surgery, thus, relenting on time. 

According to Liu, the increase in ROS production may also be responsible for SGD-induced cytotoxicity [16]. In agreement with these findings, we found that intracellular ROS production was significantly increased following SGD. Love has mentioned that ROS are found to mediate much of the damage that occurs after transient brain ischemia and in the penumbral region of infarcts caused by permanent ischemia [17] and are considered possible candidates for elucidating the pathogenesis of acute CNS injury, becoming an important therapeutic target as discussed elsewhere [18–20]. In this study, treatment with *S. litwinowii *was shown to effectively block SGD-induced ROS production, indicating that an inhibition of intracellular ROS generation might be involved in the neuroprotective effects of *S. litwinowii *thereby confirming its antioxidant role in ischemic cell injury.

To date, two major researches have been performed on *S. litwinowii*, which assessed its antitumor effects and its antioxidant properties. In the study by Tayarani-Najaran et al. the dichloromethane total extract of *S. litwinowii *was reported to have cytotoxic effects against human gastric adenocarcinoma (AGS), human cervix carcinoma cell lines (HeLa), and the human breast cancer cell line (MCF-7) and rat pheochromocytoma cell line (PC12) [8]. In another study, the CH_2_Cl_2_ fraction of methanolic *S. litwinowii *root extract was found to induce apoptosis through apoptotic pathways in human promyelocytic leukemia cells [21]. Smaller studies on the individual extract ingredients of *S. litwinowii *(e.g., wogonin and neobaicalein) were shown to be apoptogenic upon HeLa cells [22]. Neobaicalein, a flavonoid isolated from *S. litwinowii *was also found to target mitochondrial apoptotic pathways in leukemic cell lines. In this current study, the antioxidant properties of this plant were evaluated and it has been shown that *S. litwinowii *has both antitumoral and antioxidant properties. 

There are multiple ingredients within a total extract so that a serial segregation of compounds and individual assay of each can ultimately determine the exact chemical substance responsible for such effects. Wogonin and baicalein are two of many ingredients of this plant, which have been segregated so far. Analysis of each may pave the way toward a clear discovery of the true effective component. Thereafter, there will be a need to evaluate the pharmacodynamics of the drug followed by pharmacokinetic analysis, ultimately leading to drug development. 

Wogonin, Baicalein, and Baicalin from *Scutellaria* genus were all shown to possess ROS scavenging activity. These compounds also cause depletion of GSH content in human hepatoma cell lines, and therefore it is thought that the anticancer activity of these compounds may also involve a prooxidant mechanism [23]. The number of hydroxyl substitutions in the backbone structure of a flavonoid affects the antioxidant and the copper-initiated prooxidant activities. Generally, the more hydroxyl substitutions in the backbone structure of a flavonoid, the stronger the antioxidant and prooxidant activities. Flavonoids with multiple hydroxyl substitutions showed antiperoxyl radical activities even stronger than Trolox, an *α*-tocopherol analogue [24]. The possible reason why higher concentrations of *S. liwinowii* lose its protective effect against serum/glucose deprived PC12 cells may be due to the higher concentration of prooxidant compounds presented in the extract.

To conclude, the present study indicates that *S. litwinowii *treatment ameliorates SGD-induced cell toxicity in cultured PC12 cells, and its protective effects might be mediated by the inhibition of the intracellular ROS production. This study on the neuroprotective effects of *S. litwinowii *may suggest its possible application in the clinical setting to prevent and treat common neurological insults.
